# Development and validation of a MUC16 mutation-associated immune prognostic model for lung adenocarcinoma

**DOI:** 10.18632/aging.204814

**Published:** 2023-06-20

**Authors:** Honggang Liu, Tao Xin, Hongtao Duan, Yuanyong Wang, Changjian Shao, Yifang Zhu, Jiansheng Wang, Jianjun He

**Affiliations:** 1 Department of Thoracic Surgery, The First Affiliated Hospital of Xi’an Jiaotong University, Xi’an, China; 2 Department of Respiratory Medicine, Tangdu Hospital of Air Force Military Medical University, Xi’an, China; 3 Department of Thoracic Surgery, Tangdu Hospital of Air Force Military Medical University, Xi’an, China; 4 Department of Breast Surgery, The First Affiliated Hospital of Xi’an Jiaotong University, Xi’an, China

**Keywords:** MUC16, lung adenocarcinoma, immune prognostic model, prognosis, signature

## Abstract

Mucin 16 (MUC16) mutation ranks third among all common mutations in lung adenocarcinoma (LUAD), and it has a certain effect on LUAD development and prognostic outcome. This research aimed to analyze the effects of MUC16 mutation on LUAD immunophenotype regulation and determine the prognostic outcome using an immune prognostic model (IPM) built with immune-related genes. The MUC16 mutation status and mRNA expression profiles were analyzed using diverse platforms and among several LUAD patients (n = 691). An IPM was then constructed using differentially expressed immune-related genes (DEIRGs) in MUC16MUT LUAD cases, and the data were compared with those of MUC16WT LUAD cases. The IPM's performance in distinguishing high-risk cases from low-risk ones among 691 LUAD cases was verified. Additionally, a nomogram was built and applied in the clinical setting. Furthermore, a comprehensive IPM-based analysis of how MUC16 mutation affected the tumor immune microenvironment (TIME) of LUAD was performed. MUC16 mutation decreased the immune response in LUAD. As revealed by functional annotation, the DEIRGs in the IPM were most significantly enriched in the humoral immune response function and the immune system disease pathway. Moreover, high-risk cases were associated with increased proportions of immature dendritic cells, neutrophils, and B-cells; enhanced type I interferon T-cell response; and increased expression of PD-1, CTLA-4, TIM-3, and LAG3 when compared with low-risk cases. MUC16 mutation shows potent association with TIME of LUAD. The as-constructed IPM displays high sensitivity to MUC16 mutation status and can be applied to discriminate high-risk LUAD cases from low-risk ones.

## INTRODUCTION

Lung cancer (LC) is a common malignant tumor and a major contributor to cancer-related mortality globally [1]. Non–small cell LC can be classified into three subtypes: lung adenocarcinoma (LUAD), large cell lung carcinoma, and lung squamous cell carcinoma [2, 3]. LUAD accounts for approximately 40% of all LC cases, which contributes to nearly 400,000 deaths annually worldwide [4]. Tremendous efforts have been made toward the prevention, diagnosis, and treatment of LUAD, but its prognosis continues to remain poor. An increasing number of studies have suggested that the cancer malignant phenotype is influenced by the tumor microenvironment (TME) [5–7]. LC, a cancer with immune sensitivity, involves infiltration of immune cells such as eosinophils, macrophages, dendritic cells (DCs), neutrophils, natural killer cells, mast cells, T-cells, and B-cells. Nonetheless, research comprehensively investigating the relationship of immune phenotypes in the TME of LC with its prognostic outcome is scarce.

As reported in The Cancer Genome Atlas (TCGA), mucin 16 (MUC16), also named CA125, ranks third among genes with high mutation frequency and is located on chromosome 19p13.2. MUC16 becomes dissociated in the extracellular space after intracellular C-terminal phosphorylation, thereby inducing proteolytic cleavage as well as extracellular domain dissociation [8, 9]. Functionally, MUC16 has the potential to become a commonly used clinical biomarker for monitoring epithelial ovarian cancer [10]. MUC16 mutation is related to higher tumor mutational burden (TMB) and better overall survival (OS) in gastric adenocarcinoma cases [11]. Nonetheless, no study has comprehensively analyzed the relationship between MUC16 mutation and ICI response using the LUAD cohort.

The present study integrated MUC16 mutation status and mRNA expression patterns for investigating the association of MUC16 mutation with the immune landscape of LUAD. This research was conducted with an aim to analyze the effects of MUC16 mutation on LUAD immunophenotype regulation and determine the prognostic outcome. For the prognostic outcome analysis, a personalized immune prognostic model (IPM) was built with immune-related genes (IRGs) under the impact of MUC16 mutation and verified using diverse platforms and among many patients. The findings of this study showed that the as-constructed IPM is a candidate prognostic nomogram for improving the management of LUAD patients.

## MATERIALS AND METHODS

### Data collection and case screening

Information on mRNA expression patterns and somatic single-nucleotide mutation and clinical data of LUAD cases were obtained from TCGA and Gene Expression Omnibus (GEO, GSE31210 based on the platform GPL570) dataset. This research enrolled LUAD patients who had adequate clinical information. Data on some clinicopathological factors, including age at diagnosis, sex, ethnicity, MUC16 mutation status, residual tumor status, TNM stage, survival status, and survival time, were collected from the selected cases. Overall, 691 LUAD cases (n = 515 in TCGA; n = 176 in GSE31210) were selected. TCGA dataset and GEO dataset were chosen as the independent training set and validation set, respectively. Gene expression profiles and clinicopathological factors for LUAD can be obtained freely from these datasets. All analyses in the present study were conducted in strict accordance with relevant regulations and guidelines. To be specific, TCGA database was used to obtain information on somatic mutation status from 515 LUAD patients (workflow type: SomaticSniper Variant Aggregation and Masking) as well as data on RNA sequencing [RNA-seq] and clinicopathological factors from 506 LUAD cases until September 28, 2022. R software “clusterProfiler” function was used to annotate gene symbols. Overall, 386 of the 506 LUAD cases for which there was sufficient information on mRNA expression patterns, MUC16 mutation status, and clinical characteristics were screened for further analyses. After log_2_-scale transformation, RNA-seq data were further normalized using the trimmed mean of M values (TMM) function in R software “edgeR” function. When one gene had several expression levels, the mean level was used. Additionally, microarray data and relevant clinical information of LUAD cases were collected from the GEO GSE31210 dataset (http://www.ncbi.nlm.nih.gov/geo/) using the R software “GEOquery” function. R software “sva” function was used to eliminate data batch effects to conform to normal distribution, and the R software “limma” function was used for external validation of IPM’s performance.

### Gene set enrichment analysis

To analyze the relationship of MUC16 mutation with immune-related biological pathways in LUAD, gene set enrichment analysis (GSEA) was performed with TCGA data set of LUAD patients (n = 386; with MUC16 mutations) using GSEA software [12]. p < 0.05 indicated statistical significance.

### Differentially expressed genes identified according to MUC16 mutation status

R software “limma” function was used to identify DEGs in MUC16^MUT^ LUAD cases; the DEGs were then compared with those in MUC16^WT^ LUAD cases [13] upon the thresholds of false discovery rate (FDR) < 0.01 and |log_2_fold change (FC)| > 1. The MUC16-associated DEGs and the aforementioned IRGs identified in GSEA were intersected for obtaining immune-related DEGs (IRDEGs) in MUC16^WT^; these IRDEGs were compared with those in MUC16^MUT^ LUAD individuals.

### IPM establishment and verification

Adequate data were available for 386 TCGA-derived LUAD cases, including information on mRNA expression patterns, MUC16 mutation status, survival status, and survival time. The IRDEGs in MUC16^MUT^ LUAD cases and survival information of 386 patients were examined using the R software “survival” function through univariate Cox regression. DEGs were deemed to be prognostic IRGs if |hazard ratio| ≠ 1 and p < 0.05, and these DRGs were included in the subsequent analyses. R software “glmnet” function was used for LASSO Cox regression to analyze the significant prognostic IRDEGs. Additionally, the penalization coefficient was calculated to assess the model parameters’ weights. Each insignificant indicator was shrunk to zero, whereas the remaining DEGs were used to construct the prognostic risk score model. The IPM was then established by applying relevant prognostic DEGs’ coefficients: risk score = βmRNA_1_ * ExprmRNA_1_ + βmRNA_2_ * ExprmRNA_2_ + … + βmRNA_n_ * ExprmRNA_n_, where Expr represents DEG expression and β indicates LASSO Cox regression coefficient. The TCGA-derived LUAD patients were categorized as low- and high-risk groups on the basis of risk score. To determine whether the constructed IPM could distinguish patient prognosis, the difference in OS between the low- and high-risk groups was calculated and compared by constructing Kaplan–Meier (KM) curves using the R software “survival” function through log-rank test. To evaluate the constructed IPM’s predictability, time-dependent receiver operating characteristic (t-ROC) curves were plotted, and values of area under the curve (AUC) were determined using R software “survival ROC” function [14]. The same risk score median value and formula were adopted for the TCGA-derived LUAD cohort in the GEO dataset to verify the robustness of the constructed IPM.

### Prediction of immune cell proportion

CIBERSORT, a deconvolution algorithm, was first put forward by Alizadeh et al., and it can be used for quantifying cell proportions based on gene expression data [15]. To detect the proportion or abundance of 22 tumor-infiltrating immune cells (TIICs) in LUAD cases, the CIBERSORT algorithm (http://cibersort.stanford.edu/) was adopted together with LM22, a leukocyte gene signature matrix involving 547 genes for the accurate differentiation of 22 TIICs including B-cells, NK cells, T-cells, DCs, macrophages, and myeloid subsets. CIBERSORT produces a p-value for sample deconvolution by Monte Carlo sampling and measures the confidence of the result. p<0.05 indicated receivable results of TIIC proportions obtained from CIBERSORT. CIBERSORT was therefore used in combination with LM22 to quantify TIIC proportions in MUC16^WT^ and MUC16^MUT^ TCGA-derived LUAD cases, and a comparison was made between the groups. Cases with p < 0.05 in CIBERSORT were selected for subsequent analyses.

### Independence of the constructed IPM from traditional clinicopathological variables

A total of 386 LUAD patients who had available data on clinicopathological factors such as age at diagnosis, sex, ethnicity, TP53 mutation status, TNM stage, residual tumor status, and survival time were selected for subsequent analyses. Univariate and multivariate Cox regression analyses were performed to explore the independence of the constructed IPM from traditional clinicopathological factors in prognosis prediction.

### Nomogram construction and verification

Significant clinicopathological factors identified in the multivariate regression analysis were used to construct the visualized nomogram by applying R software “rms” and “survival” functions to predict patient OS at 1, 3, and 5 years. The predictability of the nomogram was evaluated by a measurement model. The bootstrap method with 1000 iterations was performed for discrimination and calibration to analyze the predictability of the constructed nomogram. To be specific, the concordance index (C-index) was calculated to evaluate discrimination, with a C-index closer to 1 indicating higher accuracy in nomogram prediction. Moreover, calibration curves were constructed to assess whether the survival predictability of the nomogram was consistent with that in real measurement; 45° C reference lines indicated the best predictability.

### RNA isolation and qRT-PCR assay

For RNA isolation, TRIzol reagent (HaiGene, Haerbin, China) was used for extracting total lung tissue RNA. cDNA synthesis kit (Takara, Beijing, China) was then used to prepare cDNA. With the prepared mRNA and primers (Tsingke, Xi’an, China), qRT-PCR assay was performed using SYBR Green PCR Kit (Takara, Beijing, China; Table 1). The 2^−ΔΔCt^ approach was adopted for determining gene expression, with GAPDH being the endogenous reference for mRNA [16].

**Table 1:** Sequences of primers.

**FKBR4**	GAAGGCGTGCTGAAGGTCAT
	TGCCATCTAATAGCCAGCCAG
**TK1**	GGGCAGATCCAGGTGATTCTC
	TGTAGCGAGTGTCTTTGGCATA
**HERPUD1**	ATGGAGTCCGAGACCGAAC
	TTGGTGATCCAACAACAGCTT
**CLEC3B**	CCCAGACGAAGACCTTCCAC
	CGCAGGTACTCATACAGGGC
**GAPDH**	GGAGCGAGATCCCTCCAAAAT
	GGCTGTTGTCATACTTCTCATGG

### Statistical analysis

R software version 3.6.3 was used for statistical analysis. Wilcoxon test was used to compare data between diverse groups. Pearson’s chi-square test was applied to determine the statistical significance level of the relationship among different variables. p < 0.05 (two-tailed) suggested statistical significance.

### Data availability statement

All data utilized in the present work can be obtained from TCGA (https://cancergenome.nih.gov/) and GEO (https://www.ncbi.nlm.nih.gov/geo/), with reference numbers of GSE31210.

## RESULTS

### Mutations in LUAD

**Figure 1:**
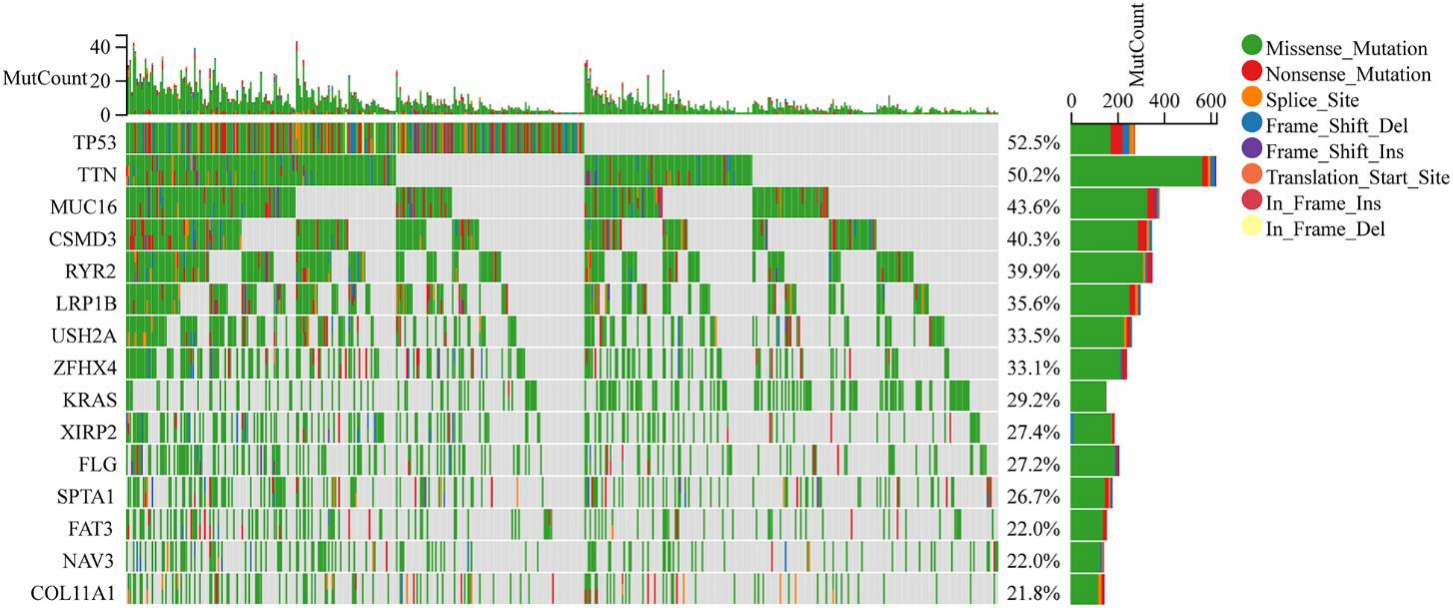
Somatic mutation landscape of lung adenocarcinoma (LUAD) patients in The Cancer Genome Atlas (TCGA) database, which was obtained from the Fire Browse platform (http://firebrowse.org).

Traditional decision-making in LC treatment is based on histological considerations. Over the past several years, treatment selection has greatly changed owing to a better understanding of tumor biology and the identification of further genetic alterations. The present research focused on identifying somatic mutations in LUAD cases. Based on TCGA data, the MUC16 mutation ranks third among all commonly occurring mutations in LUAD in terms of its occurrence frequency (Figure 1).

### DEG detection in LUAD cases with/without MUC16 mutation

**Figure 2:**
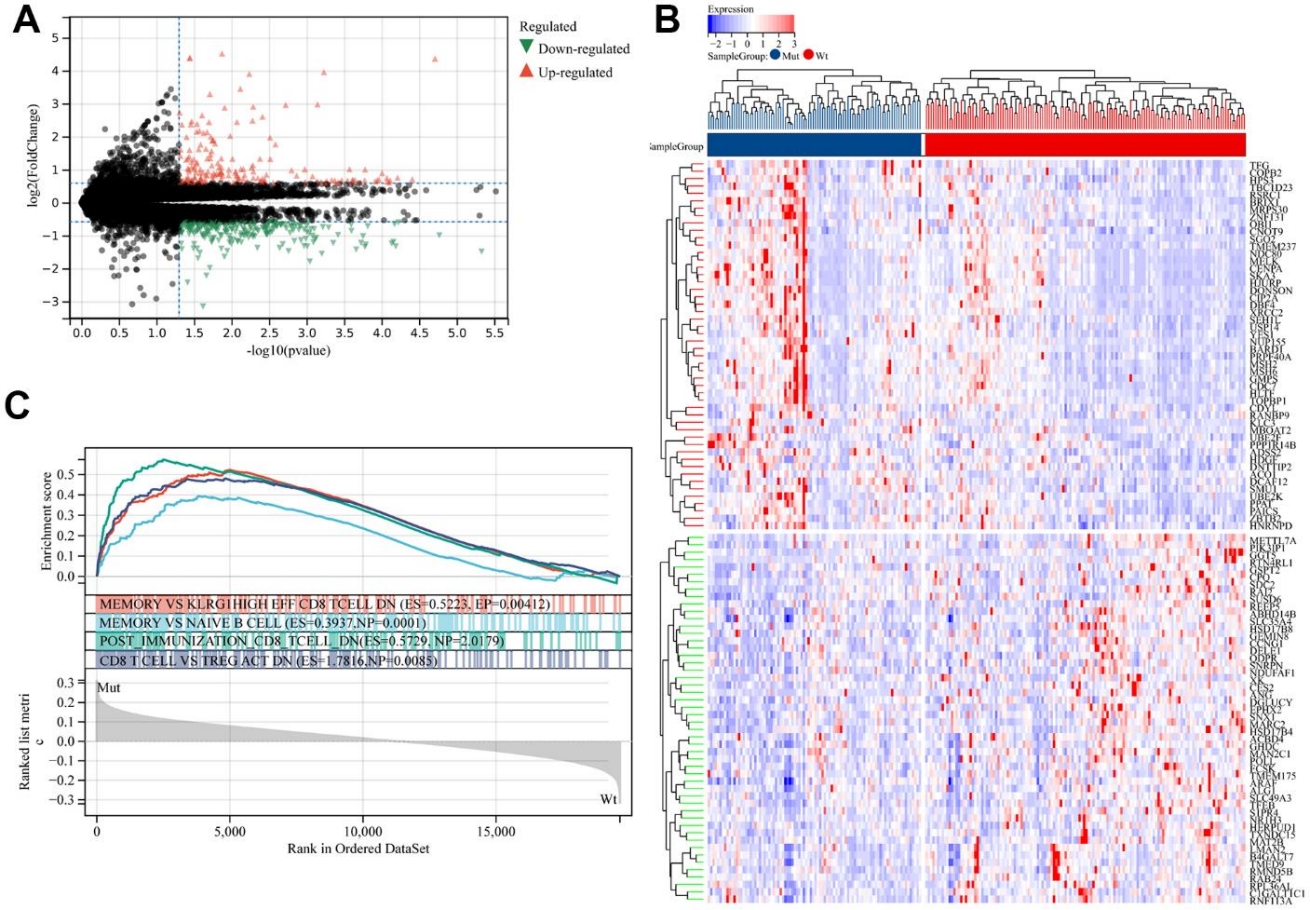
**Identification of differentially expressed genes (DEGs) in patients with lung adenocarcinoma (LUAD) with and without mucin 16 (MUC16) mutation.** (**A**) Volcano plot and (**B**) heatmap of the identified DEGs. (**C**) Gene set enrichment analysis (GSEA) of samples with and without MUC16 mutation.

MUC16 mutation had a high-frequency occurrence, indicating the tight relationship between MUC16 mutation status and LUAD development. MUC16 mutation status has been well recognized as a molecular marker for LC. Therefore, LUAD cases were classified as MUC16 mutant and wild-type (WT) groups, and the DEGs were analyzed. The results showed 794 genes with upregulated expression and 351 genes with downregulated expression (Figure 2A, 2B). For a better understanding of the functions of DEGs, gene ontology (GO) functional annotation was applied on the basis of GSEA (Figure 2C). Our results showed that genes related to MUC16 mutation status were significantly enriched in immune functions such as memory and naive B-cell, memory and klrg1 high effect CD8^+^ T-cell double negative, CD8^+^ T-cell, and Treg double negative, and post immunization CD8^+^ T-cell double negative, which indicated that genes related to MUC16 mutation status possibly exerted critical effects on immune- related processes in LUAD.

### DEG-based IPM establishment in line with MUC16 mutation status

**Figure 3:**
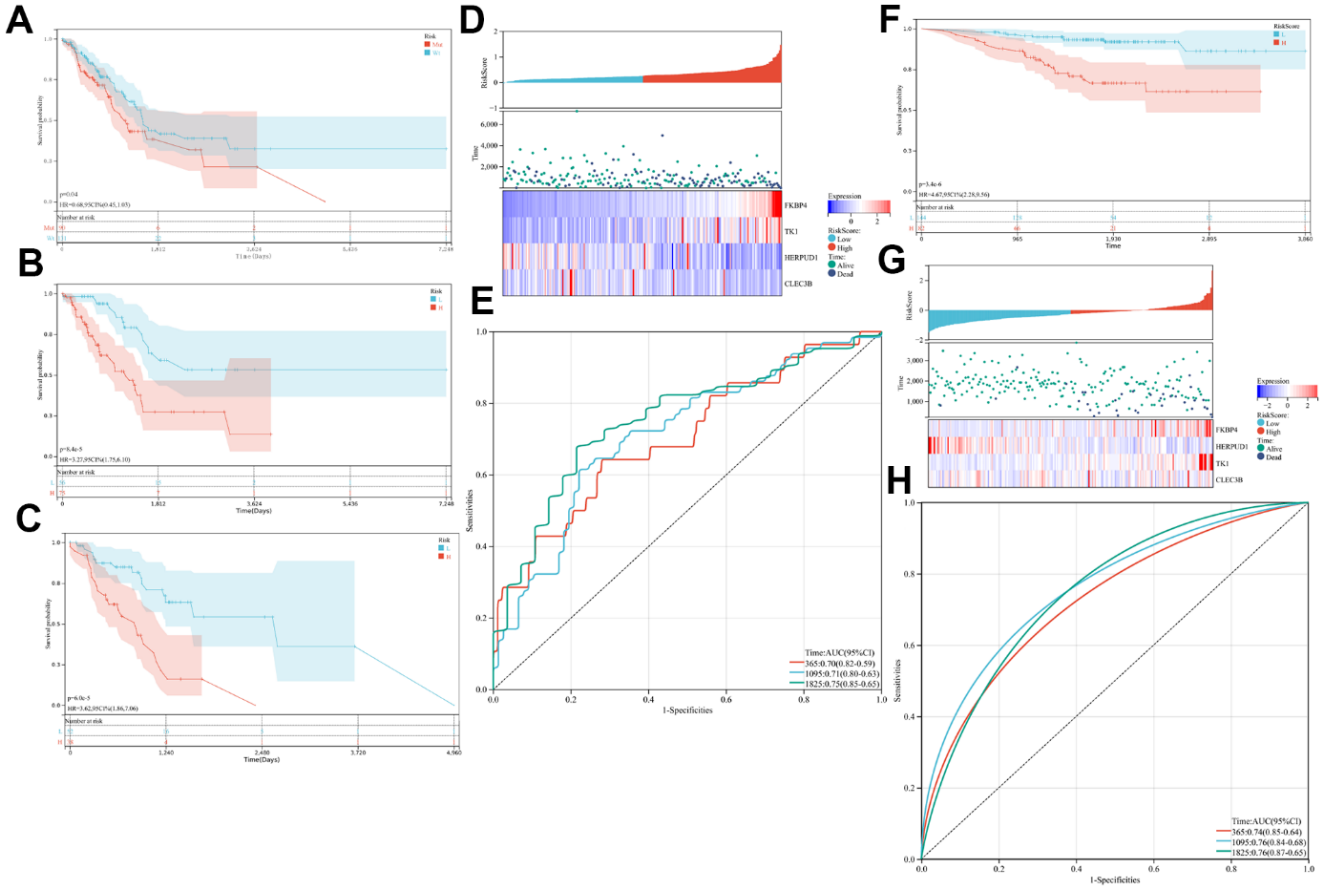
**Prognostic analysis of the immune prognostic model (IPM).** Kaplan-Meier curves of the difference in overall survival (OS) between high- and low-risk cases in (**A**–**F**) the whole The Cancer Genome Atlas (TCGA) and Gene Expression Omnibus (GEO) cohorts, (**B**) mucin 16 wild-type (MUC16^WT^) subgroup, and (**C**) mucin 16 mutation (MUC16^MUT^) subgroup. (**D**–**G**) Relationship between risk score (upper) and expression of two prognostic immune genes (bottom). (**E**–**H**) Time-dependent receiver operating characteristic (ROC) curve analysis of the IPM.

Conforming to the four-gene nomogram’s prognosis predictability, MUC16 mutation status showed a close relationship with the prognostic outcome in LUAD cases harboring mutation (Figure 3A). To investigate whether this four-gene signature, which includes thymidine kinase 1 (TK1), FK506-binding protein 4 (FKBP4), C-type lectin domain family 3, member B (CLEC3B) and homocysteine-inducible, endoplasmic reticulum stress-inducible, ubiquitin-like domain member 1 (HERPUD1), exhibited independence from MUC16 mutation status, LUAD cases were classified as low- and high-risk groups according to MUC16 mutation status. The KM OS curves for both groups, according to the constructed four-gene signature showed a significant difference between the MUC16^WT^ and MUC16^MUT^ LUAD data sets (Figure 3B, 3C). Gene expression data and risk score distribution are shown in Figure 3D, 3E shows the predictability of the as-constructed IPM based on t-ROC curves. The AUC values for the OS of our constructed model were 0.7, 0.71, and 0.75 for 1-, 3-, and 5-year survival, respectively.

### Validation of the IPM in a GEO dataset

To determine the robustness of IPM, the GEO-LUAD dataset (n = 226 LUAD cases) was analyzed to determine the constructed IPM’s performance in TCGA-derived LUAD cases. By using the same formula and threshold as those in the TCGA-derived LUAD dataset, GEO-derived LUAD cases were classified as low- and high-risk groups. Conforming to results from the TCGA-derived LUAD dataset, high-risk cases were associated with markedly poor OS when compared with the low-risk counterparts (Figure 3F). Figure 3G shows gene expression profiles and risk score distribution. The AUC values of our constructed IPM were 0.74, 0.76, and 0.76 for 1-, 3-, and 5-year survival, respectively, which were higher than those reported previously in the TCGA- and GEO-derived LUAD datasets, indicating the higher performance of our constructed IPM in short- and long-term prognosis prediction.

### TIIC landscapes between low- and high-risk LUAD cases

**Figure 4:**
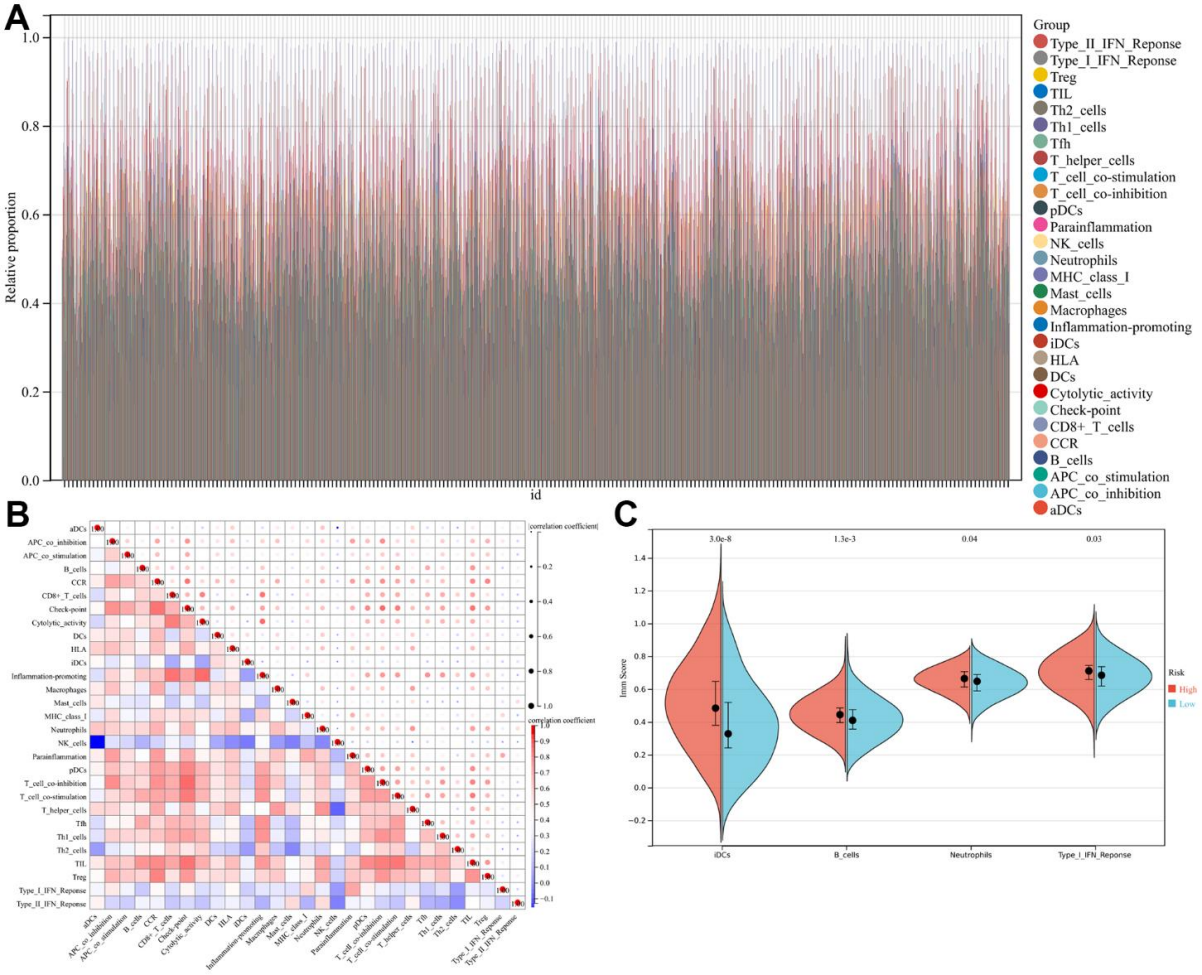
**Landscape of immune cell infiltration in high- and low-risk lung adenocarcinoma (LUAD) patients.** (**A**) Relative proportion of immune cell infiltration in high- and low-risk LUAD patients. (**B**) Correlation matrix of all 29 immune cell proportions. (**C**) Violin plots illustrating immune cells at significant proportions between high-risk and low-risk patients.

Different TIIC levels were analyzed in low- and high-risk LUAD cases. As shown in Figure 4A, the abundance of 29 TIICs showed significant differences in low- and high-risk LUAD cases. Additionally, changes in TIIC abundances possibly represented the inherent characteristic featuring individual heterogeneities. The abundance of diverse TIICs showed weak-to-moderate correlation (Figure 4B). High-risk LUAD cases showed an increased abundance of neutrophils, B-cells, immature DCs (iDCs), and type I interferon (IFN) T-cell response (Figure 4C). Different TIIC abundances seen in LUAD can serve as prognostic, predictive factors and immunotherapeutic targets, which are of great clinical significance.

### Responses to immunotherapy and chemotherapy in low- and high-risk LUAD cases

**Figure 5:**
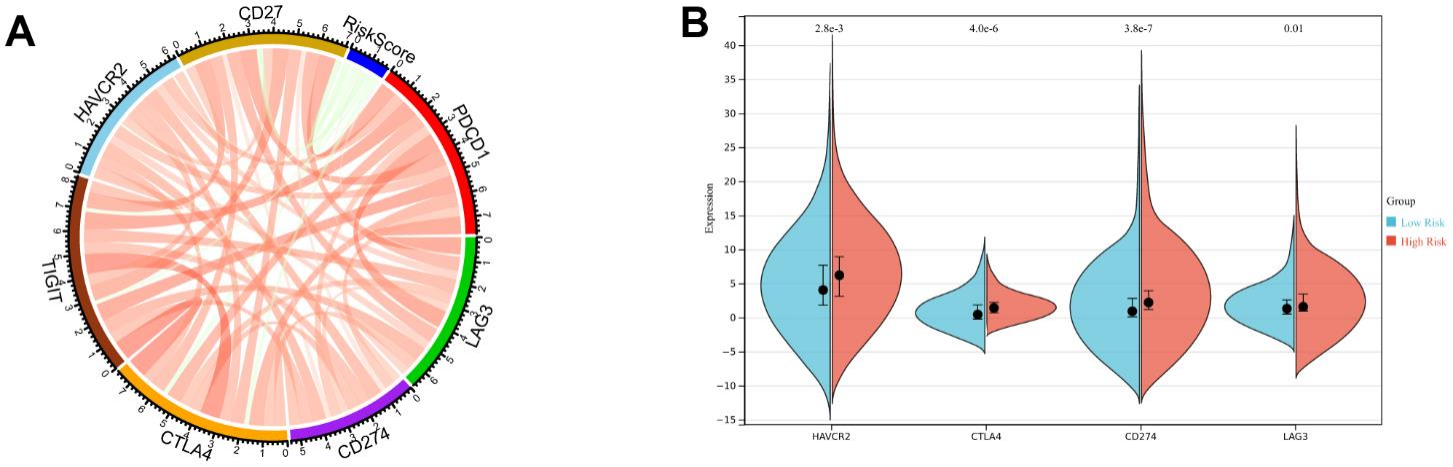
**Enrichment analysis of the immune prognostic model.** (**A**) Correlation of the risk score with the expression of several prominent immune checkpoint molecules. (**B**) Violin plots illustrating immune checkpoint molecules at significant levels between high-risk and low-risk patients.

Immunotherapy-based immune checkpoint blockade targeting PD-1 and CTLA-4 is gradually becoming the candidate treatment for different cancers. Therefore, the relationship between risk scores and key immune checkpoints (PD-1, PD-L1, CTLA-4, CD27, TIM-3, TIGIT, and LAG-3). Risk score results showed an evident relationship with PD-1, CTLA-4, TIM-3, and LAG3 levels (Bonferroni-corrected p < 0.001; Figure 5A). Furthermore, PD-1, CTLA-4, TIM-3, and LAG3 levels were analyzed in high-risk cases, and the data were compared with those of low-risk LUAD cases. High-risk LUAD cases showed markedly increased levels of PD-1, CTLA-4, TIM-3, and LAG3 when compared with low-risk LUAD cases (p < 0.05), suggesting that the dismal prognostic outcome in high-risk LUAD cases may be partially associated with the immunosuppressive microenvironment (Figure 5B).

### Changed pathways between the two risk groups

**Figure 6:**
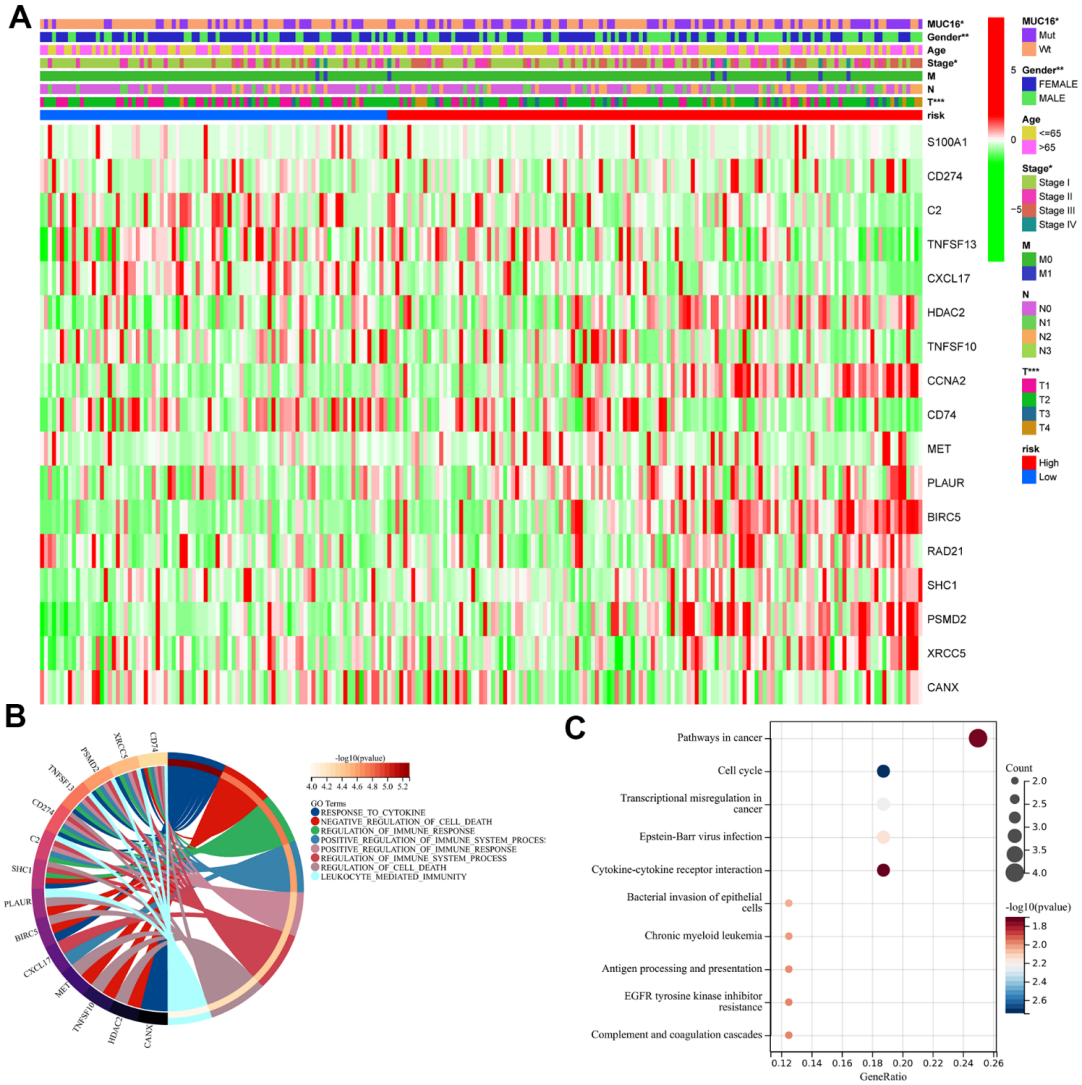
**Functional analysis of the immune prognostic model.** (**A**) Heatmap of immune-related genes that were differentially expressed in the samples of patients with high- and low-risk scores. (**B**) Circular plot of the biological processes wherein the immune-related genes are enriched. (**C**) Scatter plot of the pathways wherein the immune-related genes are enriched.

GO functional annotation was applied to provide more insights into IPM’s biological function. DEIRGs were identified in high- and low-risk LUAD patients (p < 0.05), and gene levels were related to risk scores. Seventeen IRGs were identified upon the thresholds of p < 0.05 and |Pearson’s correlation coefficient| > 0.2; Figure 6A). Subsequently, GO and KEGG analyses were performed for identifying possible biological functions (FDR<0.001) and pathways (FDR < 0.01) enriched by the identified IRGs (Figure 6B, 6C). Consequently, risk score–related genes identified from the TCGA-derived LUAD cohort were mostly associated with humoral immune response and the immune system diseases pathway.

### Associations between the constructed four-gene IPM and clinical features

**Figure 7:**
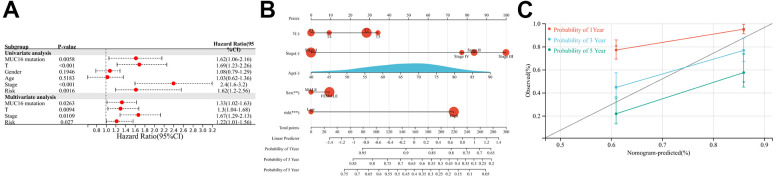
**Relationship between the immune prognostic model (IPM) and other clinical information.** (**A**) Univariate and multivariate regression analyses of the relationship between the IPM and clinicopathological features regarding prognostic value. (**B**) Nomogram for predicting the probability of 1-, 3-, and 5-year overall survival (OS) in lung adenocarcinoma (LUAD) patients. (**C**) Calibration plot of the nomogram for predicting the probability of OS at 1, 3, and 5 years.

The independence of the constructed risk score from traditional clinical factors was assessed based on the constructed four-gene IPM. The results of the univariate Cox regression analysis showed that MUC16 mutation status, risk score, T stage, and TNM stage predicted the dismal prognostic outcome of LUAD cases. In multivariate Cox regression analysis, the four aforementioned factors were identified as independent prognostic factors for LUAD (p < 0.05; Figure 7A). Furthermore, a prognostic nomogram was constructed based on the factors significant in multivariate regression. Relative to T stage and TNM stage, risk score exhibited better predictability (Figure 7B). As shown in the calibration plot, the bias-corrected line was close to the optimal curve (the 45-degree line), indicating high consistency between the predicted and observed values (Figure 7C).

### Gene signature verification with clinical samples

**Figure 8:**
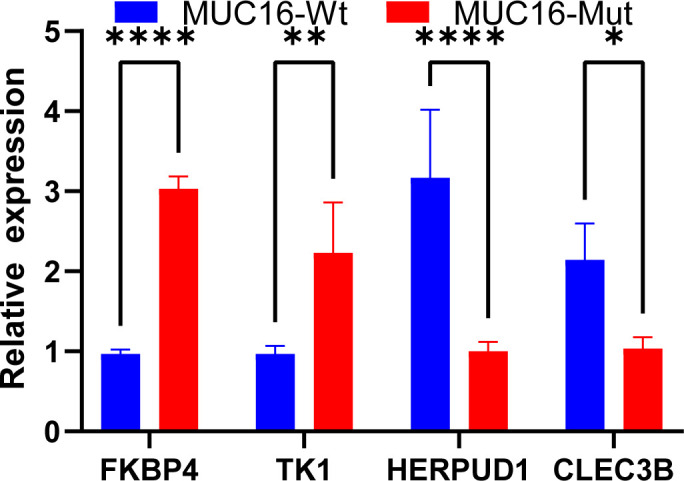
Validation of the immune prognostic model (IPM) in clinical tissue samples by performing qRT-PCR assay in lung adenocarcinoma (LUAD) mucin 16 (MUC16) mutation tissue samples and LUAD non-MUC16 mutation tissue samples.

To confirm that our gene signature was reliable, FKBP4, TK1, HERPUD1, and CLEC3B expression levels were analyzed through qRT-PCR assay of 48 pairs of LUAD tissues with or without MUC16 mutation. FKBP4 and TK1 expression markedly increased in the mutation tissue samples relative to that in the non-mutation tissue samples (Figure 8). On the contrary, the expression of HERPUD1 and CLEC3B in the mutation tissues was reduced.

## DISCUSSION

LC is a major contributor to cancer-associated deaths worldwide, and LC with MUC16 mutation causes higher mutational burden, elevated immune checkpoint protein expression, enhanced PD-L1 amplification, and increased T-cell infiltration, all of which can be regulated with the use of PD-1 inhibitors [17, 18]. Nonetheless, the mechanism of MUC16 mutation in affecting the TME and LC prognostic outcome remains unclear. Therefore, elucidating the immune impacts of MUC16 mutation status is of great importance.

The present research is the first to identify IRGs impacted by MUC16 mutation, which may provide new biomarkers to predict the prognosis and treatment of LUAD. MUC16 mutation cases were associated with dismal prognosis. Moreover, MUC16 mutation–related genes were significantly associated with immune response-related GO terms. Additionally, the four-gene IPM was constructed for predicting the LUAD prognostic outcome. According to the median risk score, LUAD cases were classified as high- and low-risk LUAD cases, with high-risk cases showing dismal OS.

In line with the tumor immunoediting hypothesis, few immunogenic tumor cells from the developed tumor can be chosen in immune-competent hosts for evading anticancer immunity [19, 20]. This approach increases the number of immunosuppressive cells (such as tumor-associated macrophages, regulatory T-cells), reduces the count of immunoreactive cells (such as follicular helper T-cells), and upregulates the expression of immunosuppressive molecules (such as CTLA-4 and PD-1) within the TME. PD-1 is an important factor regulating T-cell CD8+ exhaustion, and blocking this inhibition pathway can promote T-cell–mediated immunity in diverse cancers [21, 22]. Therefore, cases from diverse patient groups may show diverse immunotherapeutic responses. Moreover, high-risk LUAD cases exhibited an increased abundance of B cells, iDCs, type I IFN T-cell response, and neutrophils when compared with low-risk LUAD cases. Additionally, high-risk LUAD cases were associated with an increased TMB level when compared with low-risk LUAD cases, which was attributed to the higher benefits of immunotherapy [23–25]. As revealed in TIDE prediction, high-risk LUAD cases may show higher anti-PD-1 therapeutic responses. Collectively, high-risk LUAD cases demonstrated poor prognostic outcome, which was possibly associated with increased immunosuppression and decreased immunoreactivity in the TME. Such heterogeneities accelerated tumor proliferation, migration, invasion, and progression. High-risk LUAD cases could therefore gain more benefits from chemotherapy and immunotherapy.

Our study showed that MUC16 mutation status, risk score, T stage, and clinical TNM stage had a remarkable effect on OS in LUAD cases, and the constructed four-gene IPM effectively predicted the prognosis of LUAD cases. The pathological stage is an important factor in determining LC prognosis. Nonetheless, even cases at an identical stage have different clinical outcomes, suggesting that the existing classification systems may not have sufficient effectiveness to predict patient prognosis, which may not comprehensively represent biological heterogeneity in LC cases. Therefore, identifying possible biomarkers to predict patient prognosis and treat LC patients is of great importance. To the best of our knowledge, this study is the first to construct a MUC16 mutation status–related prognostic nomogram. This is a new approach for assessing LUAD cases and guiding prognosis prediction and treatment decision-making. The constructed four-gene IPM helped to differentiate prognostic outcomes in LUAD cases among diverse MUC16 mutation subtypes. Moreover, a nomogram integrating risk score and clinical information from LUAD cases was constructed to predict the prognostic outcome. Risk score showed a better performance in predicting short- and long-term LUAD prognostic outcome.

FKBP4, TK1, HERPUD1, and CLEC3B levels were measured and verified in clinical samples (tumor tissues). FKBP4 and TK1 protein expression markedly increased in MUC16 mutation tissue samples relative to that in their non-mutation counterparts. HERPUD1 and CLEC3B expression showed a contrary trend. These findings confirmed that our results were reliable.

Collectively, the present research is the first to construct the four-gene IPM based on MUC16 mutation status, which facilitates the independent prediction of LUAD prognosis. High-risk LUAD cases can gain more benefits from chemotherapy and immunotherapy. The constructed four-gene signature significantly affects LUAD management.
